# Children and Young People’s Involvement in Designing Applied Games: Scoping Review

**DOI:** 10.2196/42680

**Published:** 2023-03-16

**Authors:** Michael John Saiger, Sebastian Deterding, Lina Gega

**Affiliations:** 1 Department of Computer Science University of York York United Kingdom; 2 Dyson School of Design Engineering Imperial College London London United Kingdom; 3 Department of Health Sciences & Hull York Medical School University of York York United Kingdom

**Keywords:** serious game, game design, end user, participatory design, co-design, user involvement method, interventions, game development, children, pediatric, young people, child, youth, review method, scoping, applied game

## Abstract

**Background:**

User involvement is widely accepted as key for designing effective applied games for health. This especially holds true for children and young people as target audiences, whose abilities, needs, and preferences can diverge substantially from those of adult designers and players. Nevertheless, there is little shared knowledge about how concretely children and young people have been involved in the design of applied games, let alone consensus guidance on how to do so effectively.

**Objective:**

The aim of this scoping review was to describe which user involvement methods have been used in the design of applied games with children and young people, how these methods were implemented, and in what roles children and young people were involved as well as what factors affected their involvement.

**Methods:**

We conducted a systematic literature search and selection across the ACM Digital Library, IEEE Xplore, Scopus, and Web of Science databases using State of the Art through Systematic Review software for screening, selection, and data extraction. We then conducted a qualitative content analysis on the extracted data using NVivo.

**Results:**

We retrieved 1085 records, of which 47 (4.33%) met the eligibility criteria. The chief involvement methods were participatory design (20/47, 43%) and co-design (16/47, 37%), spanning a wide range of 45 concrete activities with paper prototyping, group discussions, and playtesting being the most frequent. In only half of the studies (24/47, 51%), children and young people participated as true design partners. Our qualitative content analysis suggested 5 factors that affect their successful involvement: comprehension, cohesion, confidence, accessibility, and time constraints.

**Conclusions:**

Co-design, participatory design, and similar high-level labels that are currently used in the field gloss over very uneven degrees of participation in design and a wide variety of implementations that greatly affect actual user involvement. This field would benefit from more careful consideration and documentation of the reason of user involvement. Future research should explore concrete activities and configurations that can address the common challenges of involving children and young people, such as comprehension, cohesion, confidence, and accessibility.

## Introduction

### Applied Games

Applied games, or serious games, describe (usually digital) games designed to drive desired cognitive, behavior, or other changes in players and the wider community [1-4]. In recent years, applied games have seen increasing interest in areas, such as *well-being* [5]*, mental health* [6]*, and education* [7,8]*.*

Applied games markedly differ from entertainment games in that they need to fit the capacities, needs, preferences, and contexts of often highly idiosyncratic target end-user audiences; find game mechanics and content that are both appealing or “fun” to the target audience; and deliver the “active ingredients” producing the desired changes, be it learning content, persuasive messages, or medical treatment regimes [4,9]. This is particularly true for applied games targeting children and young people (CYP), whose general and gaming capacities, preferences, and contexts not only drastically differ from those of adult designers and players but also from each other, depending, for example, on their developmental stage [3,10].

As in general design and development, one major successful strategy for sensitizing applied game designers to the specifics of target end users is to directly involve end users (and other relevant stakeholders) in the design, implementation, and evaluation of the game in question [11-13]. Involving users in the design process has been shown to improve use and treatment engagement [13,14], usability [15], and system adoption and adherence across stakeholders [16-18]. On the side of developers, it promises improved understanding of user needs, reduced development costs and time, and improved design quality [19]. Consequently, there are growing calls for regularly using user involvement methods in the design of applied games for health [10,11,20].

### User Involvement

However, “user involvement” describes a wide and messy field. Different research and practice communities have developed parallel traditions with confusing differences and overlaps in name, underlying values, and details of implementation, for example, human-centered design [15,21] in computing and human-computer interaction (HCI); participatory design [22,23], co-design [21], or coproduction [24] in design; patient and public involvement in health [25,26]; or action research, participatory research and science, or citizen science across the (social) sciences [15,25,27-29]. Despite regularly involving end users in the form of playtesting, applied game design still has no strong tradition of granting users more agency and input, particularly in the early stages of the design process [30,31]. In addition, recent analyses have shown that digital mental health intervention projects vary greatly in how they approach user involvement and often fail to document how user involvement methods are implemented in detail [10,20]. Bergin et al [16] observed in their recent review that few studies similarly reported any user experience of the involvement processes used—there is no even consensus on how the user experience of study participation should be captured. As a result, there is presently little empirical data on how differences in the detailed implementation of user involvement would affect end-user engagement and the overall efficacy of involvement. Consequently, we do not have evidence-based guidance on how to best involve end users and other stakeholders in the design of applied games [32], for example, for mental health [1,16,33] or education [8,34].

This lack of evidence and guidance is particularly pertinent for user involvement of CYP, and we neither know what roles, activities, or tools help engage and maintain their participation [17,29] nor what implementation factors would affect their involvement or actual adoption, adherence to, and efficacy of developed interventions (an exception was found in a study by Shah et al [35]).

### Research Questions

Thus, before we can begin to articulate potential best practice guidelines for the implementation of user involvement in designing applied games with CYP, we need basic stocktaking of actual existing practice and evidence. To this end, this scoping review aimed to describe *how user involvement has been implemented in designing games for CYP and what factors (if any) likely affect effective CYP involvement*. We articulated this aim in 4 research questions (RQs):

- RQ1: What user involvement methods are used for what purpose?
- RQ2: In what roles are CYP being involved?
- RQ3: How are user involvement methods implemented in detail?
- RQ4: What factors affect effective CYP involvement?

## Methods

### Study Design

For our scoping review, we combined a systematic literature review [36] with inductive qualitative content analysis [37]. We report our method following the revised 2020 PRISMA (Preferred Reporting Items for Systematic Reviews and Meta-Analyses) guidelines [36]. We did not preregister this scoping review because of the descriptive and exploratory nature. All study materials can be found in the Open Science Framework repository (Multimedia Appendix 1).

### Eligibility Criteria

As noted, our review focuses on *user involvement methods* in the design of *applied games* with *CYP*. With “user involvement methods,” we capture any approach that involves end users in the design of a game, such as participatory design, co-design, play-centric design, or user-centered design. With “applied games,” we mean any attempt to create or use game-based software for a nonentertainment change. “CYP” was interpreted verbatim from the records that captured a variety of age ranges, for example, 2 to 4 years or 16 to 25 years.

Unfortunately, there are no well-established standard terms or controlled vocabularies for our search focus; for example, applied games are commonly called “serious game,” “game-based,” “game for X,” or “gamified X.” Following similar reviews [10,11,38], we developed a core search string that combined a range of synonymous keywords for each aspect, such as *CYP*, *games*, *applied contexts*, and *user involvement methods* (Textbox 1).

We analyzed papers published in the last 10 years (January 1, 2010, to December 7, 2021), and as the field of applied games is quite new, we did not expect a great number of texts before this date. We implicitly focused on selecting papers that had a specified “applied context” (Textbox 1) in which, for example, games were developed with young people for “mental health” or “education.” Furthermore, we focused on the last 10 years of records owing to the development and changes in technologies and methodologies. Capturing recent (within 10 years) studies provides an up-to-date overview of the state of the literature.

We included full length papers that reported on the design of game-based software targeting CYP and explicitly featured and reflected on user involvement methods. As we are interested in understanding the factors affecting user involvement, we excluded papers that did not reflect or evaluate the user involvement methods they used. We did not exclude papers based on application contexts—we wanted to avoid unnecessary focus on a specific domain, such as health-related literature, because insights may already have been made in educational gaming or entertainment game design but ignored by us because of a narrow focus on health-related literature. We excluded short or work-in-progress papers, because they did not provide enough space for detailed reflection and reporting on the methods used. We did not investigate gray literature because of the difficulty of creating a reliable and reproducible search strategy for such a dispersed and unstructured collection of materials with no core databases. See Textbox 2 for the full inclusion and exclusion criteria.

**Full list of used synonymous key terms for constructing search strings, in which a search string combines all terms, that is, children and young people (CYP) synonyms AND games synonyms AND applied context synonyms AND user involvement method.**

**CYP**

- Young people
- Young adults
- Students
- Kid
- Child
- Adolescent

**Games**

- Game
- Video game
- Computer game
- Gamified
- Game based

**Applied contexts**

- Mental health
- Mental disorders
- Anxiety or depression
- Therapy
- Cognitive behavioural therapy
- Human computer interaction (HCI)
- Education
- Learning
- Behaviour change

**User involvement method**

- Co-creation
- Co-design
- Co-production
- Participatory design
- Patient centred design
- Patient partner involvement
- User-centred design
- Person-centred
- Collaborative
- Iterative design
- Cooperative

**Inclusion and exclusion criteria.**

**Inclusion criteria**

- Article features user involvement methods
- Article reports on the design of game-based software
- Game-based software targets audience of children or young people
- User involvement methods are evaluated or reflected on
- Full paper

**Exclusion criteria**

- No user involvement method
- No reflection or evaluation of user involvement method
- Not related to game-based software
- Not published in English
- Not a full or original paper (eg, work in progress, conference summary, or workshop)
- No children and young people involved
- Not retrievable

### Information Sources and Search Strategy

We searched 4 databases: the ACM Digital Library, IEEE Xplore, Scopus, and Web of Science. These databases mirror the interdisciplinary structure of research on applied games: the Association for Computing Machinery Digital Library and IEEE Xplore, which cover computing and HCI conferences and journals in which the bulk of technical games research is published, while Web of Science and Scopus capture disciplines such as medical research, psychology, and education.

Before starting our search, we iterated on variations of search strings implementing our target keywords (Textbox 1) for each database, because each database afforded different search strings tools. The final search strings are presented in Multimedia Appendix 2. We conducted the first full search on May 7, 2021, and the last search on July 5, 2021.

The studies retrieved from the databases were managed using State of the Art through Systematic Review (StART; version 3.3, Beta 03; Laboratório de Pesquisa em Engenharia de Software). StART identified additional studies through snowballing, which were added to the selection for screening. In addition, 14 studies were manually added.

### Selection Process

We first removed duplicate records using StART, which identified duplicates across databases. Additional duplicates were then manually removed from the selection. The first author then manually screened titles, keywords, and abstracts against the eligibility criteria; sourced full texts of the eligible studies; and then manually assessed full texts for eligibility. Finally, records that were reported in the same study were merged.

### Data Items and Collection Process

All relevant information was extracted in StART, which was then exported (xlsx format) into the qualitative data analysis software NVivo (version 12; QSR International) for open coding across the extracted information. For each eligible study, the first author extracted standard metadata (title, authors, abstract, and year) in addition to a range of descriptive data.

To describe our sample and study characteristics, the first author coded papers by the following parameters:

1. Discipline: disciplines were coded first verbatim by the title and self-description of the publication venue and then inductively aggregated; for example, a paper published in ACM HCI was coded as “human-computer interaction,” as it describes itself as “The ACM CHI Conference on Human Factors in Computing Systems is the premier international conference of Human-Computer Interaction (HCI).”
2. Date of publication: extracted from paper metadata
3. The country where the study was conducted: extracted verbatim from the Methods section
4. The number of CYP involved: extracted from the Methods section of the paper
5. The age and age range of CYP involved: extracted from the Methods section of the paper
6. The kind and number of participant groups involved: participant groups (eg, children, parents, and clinicians) were first extracted verbatim from the method sections, and then the number of different groups was counted.

To describe user involvement methods and roles, the first author coded papers for the following factors:

1. The self-labeled user involvement methods used: extracted verbatim from how the authors labeled their study in the title or Methods section; this resulted in multiple labels for some studies in which terms were used interchangeably.
2. The authors’ stated aims of user involvement: first extracted verbatim from the “goal” or “aim” statements of each paper, then inductively coded into higher-level categories, such as “determine features and functionality” or “explore methodology”; 1 paper could entail multiple aims.
3. In what capacity were CYP involved: first extracted as verbatim labels given to their roles in the paper (Multimedia Appendix 2); where roles were not explicitly labeled in the paper, we noted them as “not stated.” On the basis of the full paper description of children’s involvement, we inductively mapped each study on the Druin [39] 4-fold typology of children’s roles in the development of new technologies.

Finally, to describe *how user involvement was implemented and how to identify emerging factors affecting it*, we imported the extracted data fields and full-text PDF documents into NVivo for inductive qualitative content analysis [37]**.** The first author inductively coded the method, discussion, and conclusion sections of papers for related emerging themes in the first and second focused coding cycle [40]. Descriptively, (10) *study structure,* (11) *activities*, and (12) *media and tools* have emerged as high-level categories. Of these, the activities were clear and distinct enough that we could conduct a follow-up frequency count. In terms of (13) *factors affecting user involvement*, 4 themes emerged.

### Bias and Certainty Assessment

Because the aim of our study was narrative description, not establishing summary effects, no bias or certainty assessments were performed.

### Synthesis of Results

For descriptive summary reporting, we calculated the frequencies for (1), (2), (4), (5), (6), (7), and (11). Emerging themes and observations were synthesized through standard inductive qualitative content analysis.

## Results

### Study Selection

**Figure 1:**
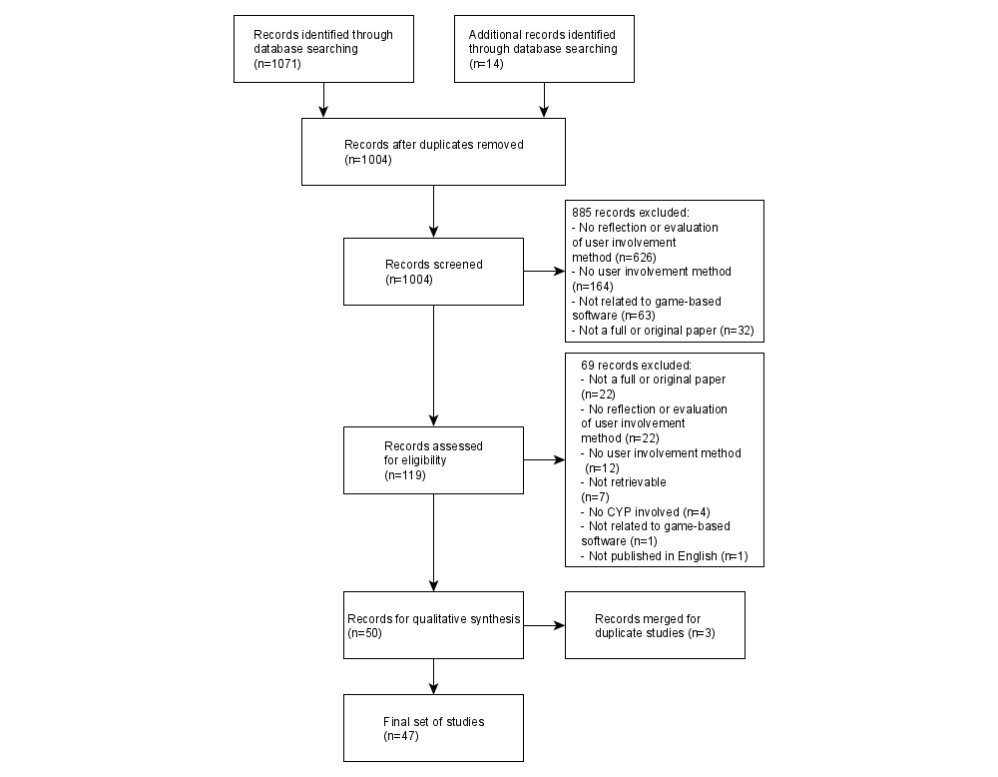
PRISMA (Preferred Reporting Items for Systematic Reviews and Meta-Analyses) flow diagram. CYP: children and young people.

Our search returned 1085 records. Title and abstract screening removed 81 duplicate records and further excluded 885 records, of which 164 (18.5%) did not clearly incorporate user involvement methods, 626 (70.7%) lacked explicit reflection of user involvement methods, 63 (7.1%) were not related to game-based software, and 32 (3.6%) were not full papers. At the full-text stage, of the remaining 13.4% (119/885) of records, we excluded a further 69 (58%), of which 22 (32%) were not full papers, 12 (17%) lacked user involvement methods, 22 (32%) lacked a detailed reflection of them, 4 (6%) had no CYP involvement, and 1 (1%) was not related to games; we could not obtain access for 7 (10%) records, and 1 (1%) was not published in English. Of the 50 remaining papers, we merged 3 (6%) paper pairs that reported the same study, resulting in 47 final studies in the analysis. Figure 1 presents the PRISMA flow diagram.

### Study Characteristics

Table 1 presents the characteristics of the included studies; 47% (22/47) of the studies were from computer science and HCI, 19% (9/47) were from education, 17% (8/47) were from serious games or games design, 11% (5/47) were from health, and 6% (3/47) were from psychology. There was no clear upward or downward trend in the publications over time. In total, 85% (40/47) of studies were conducted in the Global North, led by European countries (15/47, 32%), then the United Kingdom (13/47, 28%), Australia and New Zealand (7/47, 15 %) and the United States (4/47, 9%), followed by Brazil (3/47, 6%) in the Global South.

The sample size of CYP involved ranged from 2 to 109, with an average of 23 (SD 24.8) and a median of 13. Table 2 shows the frequency of sample sizes with 26% (12/47) studies involving 6 to 10 participants, 23% (11/47) involving 11 to 25 participants, 19% (9/47) involving 26 to 50 participants, 17% (8/47) involving 1 to 5 participants, and 15% (7/47) involving >51 participants. This is likely because (1) most studies focused on interventions for intersectional groups, such as children with learning difficulties, in which sampling can be challenging; and (2) user involvement methods often value “thick data” with small-sample qualitative methods and a struggle to scale to large participant numbers. Nevertheless, many studies have reported “small sample sizes” as a limitation.

**Table 1:** Characteristics of studies.

Study	What method or methods were labeled or framed?	Participant groups, n	Groups involved	Discipline	How long was the study?	Median age (years)	Young people, n
All et al [41], 2012	Co-design	3	CYP^a^, teachers, other experts, designers and developers, and researchers	Interaction design	Unknown	17.5	72
Alves and Hostins [42], 2019	Design-based research	3	CYP, designers and developers, and researchers	HCI^b^	4-6 months	9	4
Al-Wabil et al [43], 2010	User-centered design	4	CYP and clinicians	HCI	Unknown	15.5	6
Anacleto et al [44], 2012	Participatory design	3	CYP, clinicians, and support group	HCI	2-3 months	9.5	2
Anthony et al [45], 2012	Participatory design	4	CYP, designers and developers, and researchers	Game design	<1 day	20	12
Aufegger et al [46], 2020	Co-design	4	CYP, carers and family members, designer and developer, clinicians, and researchers	Computer science	2-3 months	9	14
Benton et al [47], 2012	Participatory design	5	CYP, teachers, and researchers	Health	2-3 months	12.5	12
Benton and Johnson [48], 2014	Participatory design	3	CYP, carers and family members, teachers, and researchers	Education	2-3 months	12	12
Bonsignore et al [49], 2016	Co-design	3	CYP, other experts, designers and developers, and researchers	Interaction design	10-12 months	15	40
Bossavit and Parsons [50], 2016	Participatory design	2	CYP and researchers	Computer science	2-3 months	13	4
Cassidy et al [51], 2015	Participatory design	6	CYP and researchers	Health	<1 day	7.5	29
Cheng et al [52], 2018	Participatory design	2	CYP, designers and developers, other experts, and researchers	Serious games	>1 year	20.5	14
Christie et al [53], 2019	Co-design	3	CYP, clinicians, designers and developers, and researchers	Interaction design	<1 day	Adolescents	30
de Jans et al [54], 2017	Co-design	3	CYP, teachers, support group, and researchers	Computer science	4-6 months	15	109
Durl et al [22], 2017	Co-design	3	CYP, teachers, support group, and researchers	Serious games	1-7 days	11.5	58
Eriksson et al [55], 2019	Co-design	4	CYP, other experts, designers, and developers	Computer science	<1 day	11	6
Gennari et al [56], 2019	Co-design	3	CYP, researchers, and other experts	Education	1-7 days	14	4
Gonsalves et al [57], 2019	Person-centered approach	3	CYP and teachers	Game design	4-6 months	10.5	46
Kang et al [58], 2021	Participatory design	3	CYP, teachers, and researchers	Health	2-3 months	9.5	7
Kangas [59], 2010	Design-based research	3	CYP, carers and family members, and researchers	HCI	<1 day	10	68
Khaled and Vasalou [60], 2014	Participatory design	3	CYP, teachers, and researchers	Computer science	2-3 months	11	13
Kostenius et al [61], 2018	Participatory design	5	CYP, carers and family members, teachers, and researchers	Psychology	N/A^c^	10.5	18
Lee et al [62], 2019	Co-design	3	CYP, teachers, and researchers	Psychology	2-3 months	13	4
Leitao et al [63], 2019	Participatory design	3	CYP, clinicians, and researchers	Education	N/A	9.5	36
Malinverni et al [64], 2014	Participatory design	3	CYP, carers and family members, designer and developer, teachers, and researchers	HCI	7-9 months	7.5	4
Martens et al [65], 2018	Co-design	2	CYP, teachers, designers and developers, clinicians, and other experts	Computer science	2-3 months	10	24
Marti et al [66], 2016	Co-design	2	CYP, carers and family members, teachers, and support group	Game design	2-4 weeks	9.5	6
Metatla et al [67], 2020	Co-design	3	CYP, teachers, and researchers	Interaction design	>1 year	10	8
Nouwen et al [68], 2016	Participatory design	3	CYP, other experts, designers, and developers	Computer science	10-12 months	18	16
Patchen et al [69], 2020	Participatory design	3	CYP, designers and developers, and researchers	Health	N/A	16.5	86
Pavarini et al [70], 2020	Participatory design	4	CYP, researchers, designers, and developers	Education	>1 year	18	30
Pollio et al [71], 2021	Participatory design	2	CYP and researchers	HCI	N/A	6.5	13
Porcino et al [72], 2015	Participatory design	4	CYP, carers and family members, and clinicians	Game design	4-6 months	9.5	6
Powell et al [73], 2019	Realist evaluation	5	CYP, teachers, and researchers	Interaction design	2-3 months	13.5	7
Rötkönen et al [74], 2021	Co-design	3	CYP and researchers	Education	N/A	9.5	5
Raynes-Goldie and Allen [28], 2014	Participatory action research	4	CYP, teachers, and researchers	Education	1-7 days	19.5	7
Regal et al [75], 2020	Cocreation or co-design	2	CYP, designers and developers, and researchers	Game design	1-7 days	Adolescents	9
Romero et al [76], 2018	Cocreation or cocreativity	3	CYP, teachers, and clinicians	Serious games	N/A	4.5	8
Stalberg et al [77], 2016	Co-design	4	CYP, carers and family members, and clinicians	Health	<1 day	11.5	54
Sutton et al [78], 2020	Co-design	9	CYP and other experts	Computer science	10-12 months	11	8
Terlouw et al [79], 2021	Iterative design	6	CYP and researchers	Education	<1 day	22	37
Triantafyllakos et al [80], 2011	Participatory design	3	CYP, designers and developers, researchers, clinicians, and other experts	Psychology	1-7 days	16	28
Vallentin-Holbech et al [81], 2020	Cocreation or cocreativity	6	CYP, other experts, designers and developers, and researchers	Computer science	7-9 months	15	35
Vasalou et al [82], 2012	User-centered design	4	CYP, teachers, and researchers	Education	2-4 weeks	10	60
Waddington et al [83], 2015	Participatory design	5	CYP and researchers	Computer science	2-4 weeks	16.5	4
Werner-Seidler et al [84], 2017	Participatory design	2	CYP, designer and developer, and researchers	Computer science	<1 day	14	21
Zhu et al [85], 2019	Co-design	3	CYP, support group, carers and family members, and researchers	Education	10-12 months	15	6

^a^CYP: children and young people.

^b^HCI: human-computer interaction.

^c^N/A: not available.

**Table 2:** Sample size variance (n=47).

Sample size groups	Frequency, n (%)
1-5	8 (17)
6-10	12 (26)
11-25	11 (23)
26-50	9 (19)
>51	7 (15)

The youngest age sampled across all studies was 3 years, and the oldest was 25 years as a part of a “young people” sample spanning ages 16 to 25 years [52]. The median age sampled was 11.5 years. Most studies (30/47, 64%) sampled an age range from 0 to 3 years (where 0 would be a precise age in years and 3 would be an age range of, eg, 6 to 9 years; Table 3).

Although our review focused on studies involving children, we were also interested in the participant groups that were involved. The plurality of studies (23/47, 49%) involved 3 different participant groups. The predominant groups were CYP (n=47, 100%) owing to inclusion criteria specifying CYP involvement. Studies also label CYP as patients, learners, or target audiences. Subject-matter experts (39/47, 83%), such as health professionals (11/47, 23%), teachers (18/47, 38%), and other experts (10/47, 21%), were cumulatively the second-most frequent participating group (Table 4). Other experts encompassed were from areas such as film, photography, art, and music. The researchers conducting or facilitating the study were the next most frequently involved group (36/47, 77%) in the design process. Researchers included those carrying out the study and references to scientists who were also involved in the study. Some studies also used designers and developers (17/47, 36%), which included game design companies, masters’ students in HCI, game designers, graphic designers, and artists, to help with the development of games or interactive technologies. Support groups (5/47, 11%) included learning assistants, pastoral care coordinators, and special education coordinators who helped support CYP participation.

Study length varied greatly, and in 17% (8/47) of studies, the study length could not be determined from the sampled records. The most frequent duration was 2-3 months (10/47, 21%), followed by studies conducted in ≤1 day (8/47, 17%). Studies conducted in ≤1 day were often short workshops or sessions of 1- to 2-hour activities. Studies conducted over 1 to 7 days (5/47, 11%) were the next most frequent. This was followed by studies that took either 4-6 months (4/47, 9%) or 10-12 months (4/47, 9%). Studies conducted between 2 and 4 weeks (3/47, 6%) and 10-12 months (4/47, 6%) were less frequent, and the lowest frequency was 7 to 9 months (2/47, 4%). A consideration to take into account is that it is not clear whether some studies are reporting the length of the whole study, including recruitment, procedure, and analysis, or whether they are reporting the duration of participants involvement in the study.

**Table 3:** Age variance (n=47).

Age range variance (years)	Frequency of studies, n (%)
0	7 (14)
1	5 (11)
2	10 (21)
3	8 (17)
4	6 (13)
5	2 (4)
6	3 (6)
7	3 (6)
8	0 (0)
9	3 (6)

**Table 4:** Stakeholder groups involved (n=47).

Stakeholder group	Frequency in studies, n (%)
Children and young people	47 (100)
Researchers	36 (77)
Teachers	18 (38)
Designers and developers	17 (36)
Health professionals	11 (23)
Other experts	10 (21)
Carers and family members	9 (19)
Support groups	5 (11)

### User Involvement Methods Used

#### Self-labeled User Involvement Method

The overwhelming majority of studies self-labeled their user involvement method as “participatory design” (20/47, 43%) or “co-design” (16/47, 34%; Table 5). However, different studies have used and understood these terms differently with no stable consensus. Some considered participatory design as the overall research area and co-design as the method [56,60,77,82]. Others positioned the 2 as separate methods [62,66], while others used terms interchangeably [75,85]. The most common definition of “participatory design” was to “involve end users in the design process” [45,48,68,72,86], which some interpreted strongly because end users fully and equally participated throughout the whole design and development process [68,70], while others read it weakly as “invit[ing] users to contribute ideas” [71]. There was further less consensus and clarity regarding the meaning and use of “co-design.”

**Table 5:** Frequency of self-labeled user involvement methods (n=47).

User involvement method labeled	Frequency in studies, n (%)
Participatory design	20 (43)
Co-design	16 (34)
Cocreation or cocreativity	3 (6)
Design-based research	2 (4)
User-centered design	2 (4)
Realist evaluation	1 (2)
Iterative design	1 (2)
Participatory action research	1 (2)
Person-centered approach	1 (2)

#### Stated Goals of User Involvement

Several studies have expressed >1 goal of involving users (Table 6). The most frequently stated aim was to design a game, either a prototype or a finished system (22/47, 47% of studies); 38% (18/47) of studies aimed to gather feedback on features and functionality; 26% (12/47) of studies were “meta”-studies aimed at examining involvement, that is, understanding the impact and form of CYP involvement overall; and a further 21% (10/47) of “meta”-studies explored a particular user involvement method or technique. For example, Benton and Johnson [48] explored the use of the participatory design approach to meet the needs of young people with autism spectrum disorder. A further 19% (9/47) of studies aimed to develop skills, meaning that the process served as a learning outcome, such as developing CYP design skills. Overall, 17% (8/47) of studies aimed to understand CYP needs and preferences around gaming, and a further 15% (7/47) of studies aimed to understand CYP’s perception and concerns around the context or technology of the study. Overall, 6% (3/47) of studies focused on improving the user experience of existing products or prototypes, and finally, 4% (2/47) of studies explicitly aimed to create guidelines on how to conduct co-design with CYP (other studies did produce recommendations or guidelines but did not state this as the intended goal behind user involvement [47,62,64,73,82]).

In summary, the included studies stated a wide range of reasons for involving users, with the overwhelming majority focusing on directly informing design and development, from formative research on user needs, preferences, concerns, and contexts (15 studies) to feedback on features and functionality (18 studies), directly making a game (20 studies), or improving user experience (3 studies). Against this stand comparatively fewer “meta”-studies in user involvement and supporting methods themselves (22 studies) and the aim to skill up CYP (9 studies).

**Table 6:** Frequency of user involvement goals (n=47).

Goals of studies	Frequency, n (%)
Design a game with participants	22 (47)
Feedback on features and functionality	18 (38)
Examine involvement	12 (26)
Explore methodology	10 (21)
Develop skills	9 (19)
Understand children and young people needs and preferences	8 (17)
Understand perceptions and concerns	7 (15)
Improved user experience	3 (6)
Create guidelines	2 (4)

### Involvement Roles of CYP

The dominant verbatim labels used for CYP involvement roles were “informant” (26/47, 62% of studies) and “co-designer” (20/47, 42%), followed by “playtester/tester” (4/47, 9%), “validation” (4/47, 9%), “co-creator” (4/47, 9%), “end user” (2/47, 4%), and “co-researcher” (1/47, 2%). Overall, 11% (5/47) of studies did not indicate a particular role of involvement (note that a single study could identify multiple roles for CYP; hence, percentage added up to >100%).

Similar to self-labeled methods, these role labels can cover a wide variety of actual degrees and types of involvement. In addition, we coded all studies using the Druin [39] influential taxonomy of 4 possible roles children can play in the design of new technology, in which each successive role can be seen to be more agentic than and encompass the previous ones: ([[[[user]tester]informant]design partner]). Each role also differs in the CYP-adult interaction (from indirect observational input to feedback, dialogue, and elaboration on adult ideas), forms of technology materializations engaged with (from ideas to prototypes to existing products), and goals of inquiry (from developing theory about use to assessing technology effects to improving design and usability):

1. *CYP as users* describes studies in which adults observe CYP using technology to assess technology effects and build use theory.
2. *CYP as testers* describes studies where CYP act as usability (or play-) testers, usually of prototypes, which can help assess effects, build theory, and improve designs; here, CYP can provide direct feedback on designs.
3. *CYP as informants* describes studies in which CYP can be involved in the full spectrum of human- or user-centered design methods, from formative research, such as interviews, contextual inquiries, and the like, to testing and validating prototypes at the evaluative end; here, CYP can engage in dialogue with adults and elaborate on their ideas and concepts.
4. *CYP as design partners* describes studies where CYP are equal stakeholders to adults; throughout the design process, they may engage in all the previously described activities, but in addition, CYP may engage in user research and design activities, such as data collection, brainstorming, or prototyping themselves, directly and jointly with adults.

We coded each included study according to these roles by examining how CYP had been involved in the procedure of the user involvement method and mapping this to the Druin [39] taxonomy (Table 7). According to this classification, in 51% (24/47) of the studies, CYP were involved as design partners; 70% (33/47) of studies involved CYP as informants; and testers and users were involved in 11% (5/47) of studies each. In 2 studies, CYP were only involved as users, and 1 study involved them only as testers. Finally, it is worth noting that 2 studies explicitly adopted Druin [39] taxonomy and ensured the involvement of CYP in each of the 4 roles [43,75].

**Table 7:** Role of children across studies.

Role	Total number of occurrences	Studies
User	5	[43,58,66,72,75]
Tester	5	[28,43,53,62,75]
Informant	33	[41-43,45,46,48-50,52,53,55-57,59,60,63,64,66-70,74-84]
Design partner	24	[22,43,44,46,47,49,51,54,55,59-63,65,68,69,71,73,75,79-81]

### How User Involvement Is Implemented in Detail

Inductive coding of the studies’ method descriptions yielded 3 high-level categories of how user involvement was implemented in detail: structure and sessions, activities, and media and tools. *Session structure* describes recurring stages and an overarching facilitation organization. *Activities* captures the specific tasks conducted with stakeholders, while *media and tools* describe the range of media and existing games used in activities.

#### Session Structure

In total, 8 studies involved an initial *onboarding or sensitizing stage* to create familiarity with the project topic and team, communicate its goal, and help in understanding the upcoming process [22,50,54,68,74,75,78,81]. This was suggested to build trust between participants, foster user engagement, and make an effective co-design process more likely [22,81]. In 2 studies, onboarding consisted of exploring an existing solution to familiarize oneself with the technology and underlying concepts of the research area [48,81]. In another study, the sensitization steps involved introducing users to the topic through challenges and competitions, which also helped build trust with facilitators [22].

Ten studies [41,42,44,47,48,59,63,64,82,85] described an *ideation or brainstorming stage* to help frame user needs and provide users with a starting place. Notably, brainstorming could be used to generate game ideas [42] or to understand existing user practices and areas of technology-based support [44]. This was often supported by starting exemplars and paper templates [41,47,59,63,64], such as empty scenario storyboards or empty mobile phone screens, or a homework task and prepared video-seeding material for ideas [50]. Two repeatedly mentioned challenges and considerations for this phase are the common “groupthink” converging of participant groups on a first or loudly voiced idea blocking further ideation [47,48] and CYP capabilities to actually conduct ideation [47]—although some noted that CYP tends to bring a beneficial high degree of gaming literacy [82].

In total, 17 studies [45,47,49,51,52,55,56,58,62,63,65,70,71,74, 75,77,79] reported the *prototyping stage*. These prototyping stages included sessions in which CYP were involved in the design and development of game ideas [52,58,75], game characters or narrative [49,62,71], and generated alternative ideas to an existing idea [63,77]. Prototyping was seen to afford a sense of ownership in the resulting designs for CYP [62], often challenging researchers’ assumptions on end users, and often leading to in-depth reflection from participants [55,70,71]. Reflection was 2-fold, in which studies designed sessions of reflection, and reflection surfaced unexpectedly. For example, 2 studies designed reflection meetings to iterate and improve future design sessions [45,74], whereas another study found that the design process led to participants reflecting on their game-play experiences compared with other participants [55]**.** One study deliberately scheduled reflection meetings after prototyping as the basis for future iterations [74]. Two challenges observed in this phase were the limitations of the prototyping tools used and what CYP wanted to portray, in which the limitations of paper prototyping were challenging for CYP to articulate the actions they expected from a digital prototype [51].

An *evaluation stage* was rarely mentioned across studies, where the design process was discussed with participants on its engagement, effectiveness, and efficacy as a user involvement method. Outside the reflection meetings, 4 studies scheduled an evaluation stage to gather feedback on the user involvement process [45,47,80,81]. These studies discussed how to make the interaction during the design process more engaging [45], how they found the process as a whole [80], how they could be more involved [47], and how they could cooperate with others [81]. Notably, only 7 studies evaluated the prototypes developed at the end of the study [43,45,47,52,53,80,81].

#### Activities

Our coding resulted in 45 different activities, of which 19 (42%) were only shown in a single study (omitted in Table 8). Paper prototyping was the most frequently conducted activity, mentioned in 20 studies with labels, such as “paper play activity” [44] or “sketchbook prototyping” [52]. We coded this separately from low-fidelity prototyping (used in a further 7 studies); even though the boundaries between the 2 are not clear-cut, some low-fidelity prototyping would involve paper storyboards and sketches. Regardless, both were reported as affording a positive experience to end users [58,68,80], for example, by giving every participant some hands-on experience [45]. Hands-on experience affected both agencies, in which hands-on experience was a method to assess games with users and understand outcomes [65,66], and learning, in which hands-on experience served as a method of learning technology or understanding the context [28,45,59]. The prepared templates were repeatedly mentioned to facilitate prototyping [63,86]. Paper prototyping was reported to be inclusive [45], low cost [51,65], and using only easily accessible materials [51]. Observed challenges in prototyping included struggling to represent the intended playful interaction with digital technology [51], and that was less suited to older teens because of the hypothetical or “blue-sky” situations when they are at a developmental point of building their own opinions distinct from others [49].

Mapping between activities and design stages was difficult because activities were used across different design stages and in cases that were not transparent when and why activities were used. For example, (focus) group discussions, the second-most prevalent activity (19 studies), were used as icebreakers [45,56], to generate ideas [46,50,63] and reflect on the end product [22,55,57,61]. In another instance, the fourth-most prevalent activity interviews (used in 17 studies) were used equally formatively to discover and define the problem space [41,59,82] and evaluate prototypes or concepts [24,52,53,81]. Presumably because our study sample overall leaned toward “earlier” sensitizing, ideation, and prototyping stages, most activities were used in these stages; only *feedback sessions*, some instances of *game-play evaluation*, and 1 *timeline activity* [68] (asking CYP players to chart their game-play likes and dislikes and experiences of challenge) occurred during an evaluation phase.

The included studies entailed little explicit reflection or evidence of the effectiveness of the conducted activities, with a few exceptions. For example, Nouwen et al [68] outlined which particular activities generated particular user insights and related design impacts. Pavarini et al [70] organized feedback sessions in which CYP could suggest features and processes for better future user involvement. However, even these observations remain unvalidated and can disagree with one another. Thus, although several studies recommended “free play” to provide CYP creative freedom of expression [55,68,86], Nouwen et al [68] found that this had little design impact because the media created during free play were unsuitable for the design brief. Further details of methods and activities can be found in Multimedia Appendix 3.

**Table 8:** Frequency of activities across studies that were used in more than one study (n=47).

Activities	Description	Frequency, n (%)
Existing games (demonstration or play)	Participants are given existing games, game prototypes, or materials to play or interact with	18 (38)
Group discussion or focus group discussion	Participants work together and talk about ideas, concepts, or solutions	19 (40)
Paper prototyping	Participants use mainly paper to design ideas and solutions	20 (43)
Interviews (with end users)	Researchers interview end users (CYP^a^) for their thoughts, preferences or feedback on a prototype, context, or problem area	17 (36)
Playtesting	Participants are given a prototype game and asked to play it (feedback is optional sometimes)	14 (30)
Storyboarding	Participants are given panels, comic strips, and slides and asked to generate how they would use the game, a scenario, or a solution	11 (23)
Surveys or Questionnaires	Both qualitative and quantitative surveys. Includes tools such as Likert scales and researchers directly asking CYP to give an answer on a scale	12 (26)
Brainstorming	Participants work together to generate ideas through group discussions, activities, or in some cases, individually	9 (19)
Role-play (or simulation)	Participants take on the role of others, for example, CYP acting as doctors, to bridge the challenges different stakeholders undergo	9 (19)
Feedback Session	Participants are shown a prototype and asked to give feedback	7 (15)
Introductory media (presentation, movie, etc)	A presentation, movie, talk, or activity is used to help onboard young people on what the goal of the workshop or activity is	6 (13)
Low-fidelity prototyping	Prototyping with models, figures, blocks, and early development digital games	6 (13)
Free play	Participants are given a game or demo and no goals are set; they can interact how they choose	5 (11)
Sticky notes	Tasks involving sticky notes or post its, usually a group-driven task	5 (11)
Scenario-based tasks	Participants are given a scenario in relation to the applied content with which to design a solution	4 (9)
Blank template task	Participants are given a template, for example, the wireframe of a phone screen, and use these to design solutions	3 (6)
Game idea or feature evaluation	Specifically when participants evaluate a component or proposed idea of a game	3 (6)
Logbooks (or taskbook or diaries)	Participants complete a workbook or taskbook after activities; also includes participants keeping a log of their behavior	3 (6)
Storytelling	Users create and share stories or concepts	3 (6)
Timeline (narrative design)	Creating a journey, or series, of steps in which a procedure, process, or story occurs over time or between different users	3 (6)
Game design workshops	Workshop that encompassed ideation and then evaluation and reflection of those ideas on how they can be improved	2 (4)
Icebreakers	Informal discussions and activities for participants to get to know each other	2 (4)
Map task	Using real world maps to choose a setting for their game idea and generate ideas [41,66]	2 (4)
Peer interviews	Interviews conducted by CYP to other CYP to gather data and help understand the context area	2 (4)
Question and answer	An activity were users presented predetermined answers (eg, cards) to questions proposed by researchers	2 (4)
Sensitization session	“Packages” or information circulated before user involvement sessions to understand CYP experience with context and games	2 (4)

^a^CYP: children and young people.

#### Media and Tools

Following the study by Brandt et al [87], participatory design tools and media can be classified into whether they support the practices of *making*, *telling*, and *enacting*. Bossavit and Parsons [50] observed that 2D mapping (making tool) and videos (telling tool) were useful for understanding concepts, while playing games (enacting) supported idea generation.

The choice of the media or tools used was a point of contention. Some suggested that CYP struggled to express themselves speaking while at the same time preferred speaking to writing [45,47]. In comparison, visual approaches and working with visual aids proved more engaging and effective, especially in younger age groups, potentially owing to their less-developed reading and writing abilities [49,50,62,66]. An exception to the method of media was seen in the studies by Metatla et al [67] and Regal et al [75] with visually impaired children, in which educational robot toys and physical building blocks were used to facilitate creativity through touch and sound. Overall, it seems that preferences for spoken, written, or visual media are related to the age of participants [45,49,62]. Therefore, this suggests that the choice of media and tools used should be determined by understanding participant preferences before the activities take place.

In total, 18 studies used existing games as activities. Existing games varied between existing prototypes developed before the study and existing commercial games [71,76,88]. Existing games were used not only as a tool for narrowing the scope of the research and “managing the expectations” of young participants but also as an icebreaker activity [76,88]. Existing prototypes were sometimes used as a starting point for prototyping or were introduced to users for feedback [45,47,76]. That said, several studies found that concepts and features proposed by CYP were usually informed by the commercial games they were familiar with [50,60,82,85].

### Factors Affecting User Involvement

#### Overview

In this section, we report 5 prominent themes that studies have repeatedly covered as affecting user involvement: *comprehension, cohesion, confidence*, *accessibility,* and *time constraints*. We intentionally did not tie these themes to one particular conception or standard of “good” involvement. Rather, we took the study authors’ own conceptions at face value: if a study articulated something as problematic or positive, we took it as such, also to reflect the variety of goals studies articulated for user involvement.

#### Comprehension

This theme captured that CYP *regularly struggled to understand the design process and context they participated in*. CYP repeatedly reported that they did not fully comprehend or remember the benefits or outcomes of the project and how it would impact their daily life [44] nor the aim or purpose of the task assigned to them [72,77,81]. A usability evaluation of one involvement method similarly revealed that tasks were either hard to identify and understand or hard to perform [77]. This problem could be even more common, as Waddington et al [83] observed that CYP did not mention when they struggled with an activity or prototype in their study, so long as they could engage with it; they therefore found it often necessary to gather additional external stakeholder feedback, because CYP did not complain. According to Porcino et al [72], some of these comprehension issues could arise from the lack of a clearly stated objective, insufficient time for a task or technology to become familiar with it, or facilitators not familiar with a given technology or method.

Relatedly, *how well-informed CYP were about a subject matter* impacted the efficacy and efficiency of user involvement: participants who were familiar with a given context proved to be more productive and gave much more concise feedback than unfamiliar ones [53]. Similarly, studies have found it difficult to define problems and identify designs in areas where participants were ill-informed about [54,81]. This should not be considered a one-way street. For example, Durl et al [22] found that involving vulnerable adolescents in a co-design study on alcohol abuse not only resulted in better design results but also made the adolescent participants more informed about alcohol abuse.

A final challenge to comprehension was *lacking familiarity with used technologies and methods*: this could be the use of basic technology like microphones [74] or the fact that participatory methods differ strongly from what CYP may be used to do in adult-guided activities in school. CYP expected asymmetry in power with adults dictating the direction of the study but experienced more symmetry in power and decision-making because of their suggestions being acknowledged [81]. This challenge could be addressed with additional introductory training and warm-up activities, which consume additional money and time [60].

#### Cohesion

Most studies involved ≥3 different groups of stakeholders. Therefore, it was unsurprising that many studies reflected on *different groups working together effectively as a united team* as a major factor of effective user involvement, which we here will call *cohesion*.

First, several studies reported *struggling to achieve agreement on a concept, solution, or decision*, likely because of the control each stakeholder group was given [42,74,76]. Control in terms of decision-making and contributing to the design process could be caused by expecting CYP to come to a natural agreement without clear constraints or guidance from adults. Two studies in particular presented open questions to young people and then presents “no right answer,” which could be the cause of friction between young people [74,76]. Common approaches to this issue were discussion (for understanding different points of view) and voting mechanisms (for integrating disagreeing points of view into a decision) [76]. A connected challenge was ensuring that different end-user groups contributed more or less equally to decisions and end results [42,50,64,65]. Several authors observed that cohesion required *trust* between participants, although no particular approach has been suggested to build trust [42,76].

Another key factor for cohesion was a *cooperative and collaborative mindset and atmosphere*. Seven studies noted that these allowed CYP to express their views, support each other, and share ideas [22,28,42,55,59,80,81]. In contrast, a competitive dynamic was found to result in less-open idea sharing [76]. The study by Triantafyllakos et al [80] was one of the only studies to reference incentives, in which they used competition and challenges among participants to generate more ideas.

Breakdown in *communication* could lead to frustration and disappointment [76]. So, how can we afford to function in communication? One study found that a “natural” flow of communication among CYP in which each would have a chance to speak required dedicated facilitation [74], whereas another found that CYP compared with adults were much more direct and unfiltered in criticism, suggesting adult stakeholders should capitalize on this and not be guarded or protective of their ideas [47]. Using existing examples of games [45], as well as explicit tools and conceptual frameworks [55], was found to facilitate discussion.

#### Confidence and Empowerment

*Confidence* describes CYP’s beliefs in their ability to effectively participate in user involvement, akin to the psychological construct of self-efficacy [89], and influences their participation in several ways [43,68].

CYP would participate more deeply if they felt more confident, which was largely seen as grounded in their past experience; for example, CYP’s past experience with film production or game-making [81], using existing technology CYP are familiar with [44,85], immediate past experience of progress in particular workshop activities [68], or simply longer participation over time [47] would all increase CYP confidence, with positive effects.

However, studies differed in their view of whether CYP are generally confident to voice their opinions [43,83]. Two studies found that CYP had no apparent issue voicing their views directly [47,53], whereas others suggested to use behaviorally “honest signals,” such as eye tracking, because CYP aged 5 to 12 years may say what adults like to hear [43,76] or observed parental interference as an obstacle to CYP sharing unfiltered feedback around sensitive topics like sexual health [62]. Flexibly adjusting group sizes and session lengths to fit CYP needs was found to make them more comfortable overall and share their views more openly [86].

*Empowerment* was also discussed in studies where young people were given control to make decisions or choices, facilitated by participating in activities, ownership of end products, and the innate challenge of designing a game, all contributed to a sense of empowerment [45,61,81]. While giving CYP power to make decisions over the end product increased a sense of empowerment [77,86], multiple and repetitive user involvement activities were reported to diminish it over time [64]. In addition, there were concerns about the misuse of empowerment as a method of manipulating participants to support the findings desired and empowerment tokenism, where the control over things lacks importance and others make important decisions [61]. Treating CYP as true design partners in game design was found to make it easier for adult participants to connect with CYP’s concerns and, in turn, foster creativity [28].

#### Accessibility

*Accessibility* describes whether the CYP felt the involvement process was accessible to them and included them in the design. Accessibility was mentioned as a concern, especially in studies that targeted an end-user CYP population with specific needs that forced shaping user involvement around them [64,74,79,83]. Tailoring involvement tools for CYP’s varying abilities [50] and engaging experts associated with CYP’s disabilities (such as carers or teachers) [67] were 2 proposed strategies for improving accessibility. One game-specific accessibility issue mentioned was the development of game mechanics that would be accessible to all players, including those with disabilities, but remains challenging for all end-user groups [74,83].

#### Time Constraints

Time constraints have emerged as a major consideration in structuring user involvement. Co-designing games is time-consuming [65], to the point where de Jans et al [54] suggested that the months required for adults and CYP learning how to collaborate does not fit industry game development timelines. Several studies found that practical time constraints resulted in insufficient discussion of all ideas [74], insufficient preparation, and insufficient time for producing deliverables [76]. Three studies reported breaking the design process into separate phases across different days or sessions to maintain interest, attention, and energy as another important time-related constraint 51,75,76

## Discussion

### Principal Findings

Out of a total of 47 studies, 36 (77%) of our sample were self-labeled participatory design or co-design (Table 5). In line with previous reviews in related fields, such as co-design in health [90] or CYP involvement in child-computer interaction [91], these 2 broad labels gloss over a wide variety of actual involvement roles, aims, and activities. Most studies sampled (35/47, 74%) involved CYP as “mere” informants and about half (24/47, 51%) as true design partners (Table 7). These numbers appear to indicate deeper user involvement for CYP than in recent systematic review of general serious game development processes by Maheu-Cadotte et al [92] (who found only 1 in 21 processes involved participants as co-designers). However, because their inclusion criteria did not limit eligible studies to those with explicit user involvement, this is an unequal comparison.

The stated aims for user involvement ranged from instrumental ones of making a game or identifying features and functionality to meta-methodological interests, upskilling, and many others. We counted 45 different activities, with prototyping, interviewing, playtesting, and playing existing games being the most common. Studies commonly reported “early” onboarding, ideation, and prototyping phases; we found little mention of “later” development or production stages [64,78,82]. Furthermore, no records linked or referenced openly shared user-created design materials outside of screenshots and workshop photographs. Studies used a wide variety of tools and media, with some consensus that these should be tailored to the (developmental) preferences and abilities of the involved CYP. The reviewed studies did not discuss users’ accreditation or rewards for their contributions, outside of 1 example, which suggests uncertainty on how to motivate or reward CYP for their participation.

Our qualitative coding of method reflection produced five factors that are likely to affect successful CYP involvement:

1. Comprehension: the better the CYP know and understand the context and subject matter and design process and used methods, tools, and technologies, the better their engagement and results seem to be.
2. Cohesion: engaging and effective CYP involvement depends on different stakeholder groups working collaboratively as one team, with reaching an agreement as a common challenge and facilitating a comparative noncompetitive mindset as an important success factor.
3. Confidence: the more confident the CYP were in their ability to participate, license to participate, and agency granted to them, the deeper they seemed to engage.
4. Accessibility: both age and disabilities or special needs put extra demand on making user involvement accessible to all participating CYP.
5. Time and space: affording creative time and space can help further the discussion of ideas and maintain interest throughout participation.

### Reflection

Perhaps the strongest overarching observation when conducting this systematic review was how differently studies understood and used relevant terms such as “co-design” (2 studies could label their activity “co-design” and “focus group” yet do drastically different things); how differently and unevenly studies reported methods; how differently studies implemented these methods; and in how little detail studies actually documented their involvement methods, despite the fact that our review systematically sampled studies that explicitly reported and reflected on their user involvement. For instance, although we tried to map activities onto “where” in a standard design process, following the example in Vandekerckhove et al [20], we were not able to do so because of the low to absent detail of reporting. This is sadly in line with prior findings on co-design methods: studies rarely report sufficient detail to reproduce them, and there is no standard reporting format [90]. This lack of consensus terminology and reproducible method documentation standards hinders replication. This makes systematic assessment and integration of evidence difficult. In addition, it impedes actual know-how flows beyond tacit and in-person sharing between the members of a research group and project.

Unless we have clear, reliable, and reproducible standards for identifying and reporting types or degrees of user roles and identifying and reporting involvement methods, it is difficult to generate a meaningful systematic body of evidence on which roles or methods work better or worse for which groups of participants, contexts, or aims. This may be one reason why only 4 of the sampled studies reported any qualitative empirical evaluation of what worked and what did not with their involvement methods, even though 46% (22/47) of studies stated meta-methodological aims, such as assessing end-user involvement or involvement methods. Consequently, most reflections and derived recommendations on how to conduct user involvement remain speculative. Thus, we cannot conclude with much certainty anything about, for example, which user roles might work “better.” Although there is a normative preference for the “deepest” possible involvement in much of participatory design, several studies in our review suggested that CYP struggled to act as meaningful co-designers in applied game design [76] and were more able to express themselves as play testers than when sketching their own concepts [63]. This may be partially because of the fact that applied game design requires both domain and game design expertise, which CYP likely does not bring, which may be bridged by more careful onboarding activities or involving multiple stakeholder groups with complementary expertise [60]. Although the involvement of young people has been reported to be essential to realize game prototypes, whether CYP has expertise, or even the availability to consistently collaborate, is a challenge for future research [93]. The important point here remains that we are at present not able to answer such questions or advise on “what works best” because of poor reporting.

### Limitations

Our sample is obviously limited by its English-only focus and 10-year date range. The literature before 2010 would potentially paint a distinctively different picture, which would lead to divergent conclusions in this review. Study selection and coding were all performed by the primary author alone, meaning that we could not quantify the likely reliability and reproducibility of these steps, for example, intercoder reliability. The overall poor quality of method reporting in the sampled studies and the lack of existing controlled vocabularies or taxonomies of involvement methods means that our coding involved substantial degrees of judgment and construction in categorizing different kinds of involvement activities or classifying studies by user role. This does not impact verbatim extractions of self-labeling or similar, and we believe our overall assessment of the sheer diversity of reported practices is not touched by this.

### Future Research

The first obvious direction for future work is to provide a robust shared basis for reporting and assessing user involvement in applied game development (with or without CYP); we need better consensus methods for defining, labeling, and identifying user involvement roles and methods and better guidance and standards on reporting user involvement in sufficient detail for later analysis or replication. Second, on this basis, we can start to analyze and empirically evaluate what kinds of session structures, methods, roles, media, and tools are more engaging in CYP and more effective for different involvement aims. Third, our review suggests that comprehension, cohesion, confidence, accessibility, and time constraints are likely to impact CYP involvement. Here, methodological research can explore whether and how much these factors matter, and then again, which structures, methods, roles, media and tools, and specific implementations thereof are better suited to support them.

### Conclusions

This scoping review aimed to explore how CYP have been involved in the design of applied games. We found that a small range of labels (co-design and participatory design) hid a wide variety of actual involvement methods, aims, structures, roles, and implementations. Comprehension, confidence, cohesion, accessibility, and time constraints emerged as 5 likely nonexhaustive factors affecting effective and engaging CYP involvement. However, the reviewed literature documented its user involvement practices inconsistently and in little detail, and its recommendations for future practices are largely not grounded in robust empirical evaluations of (alternative) involvement approaches. Future work is needed to advance more robust and reproducible documentation of user involvement to enable knowledge sharing, as well as more systematic research on “what works” in user involvement of CYP in applied game design.

## Appendix

Multimedia Appendix 1 Open Science Framework repository.

Multimedia Appendix 2 Search strategies.

Multimedia Appendix 3 Definition of methods labeled and activities.

## Abbreviations

CYP: children and young people
HCI: human-computer interaction
PRISMA: Preferred Reporting Items for Systematic Reviews and Meta-Analyses
RQ: research question
StART: State of the Art through Systematic Review
