# Supplementary material for: Children and Young People’s Involvement in Designing Applied Games: Scoping Review
Source: JMIR Serious Games. 2023 Mar 16;11:e42680. doi: 10.2196/42680 (PMC10131627; doi:10.2196/42680)
Supplement: Multimedia Appendix 1 [file games_v11i1e42680_app1.docx]

Multimedia Appendix 1

Data and documentation relating to this review can be accessed here <https://osf.io/ghpfr/>. There are txt files to the search strategies, StARt extracts and codebooks which can be accessed.
