# Supplementary material for: Children and Young People’s Involvement in Designing Applied Games: Scoping Review
Source: JMIR Serious Games. 2023 Mar 16;11:e42680. doi: 10.2196/42680 (PMC10131627; doi:10.2196/42680)
Supplement: Multimedia Appendix 2 [file games_v11i1e42680_app2.docx]

Multimedia Appendix 2

| ACM Search String |
| --- |
| [[Publication Title: cocreat*] OR [Publication Title: co-creat*] OR [Publication Title: codesign*] OR [Publication Title: co-design] OR [Publication Title: particip*] OR [Publication Title: "participatory design"] OR [Publication Title: patient*-cent*] OR [Publication Title: patient*-partner*] OR [Publication Title: co-produc*] OR [Publication Title: coproduc*] OR [Publication Title: user-cent*] OR [Publication Title: collaborat*] OR [Publication Title: cooperat*] OR [Publication Title: co-operat*] OR [Publication Title: iterat*]] AND [[Publication Title: game*] OR [Publication Title: "video game*"] OR [Publication Title: videogame] OR [Publication Title: "computer game*"] OR [Publication Title: gami*] OR [Publication Title: game-based]] AND [[Abstract: adolescent] OR [Abstract: child*] OR [Abstract: "young people"] OR [Abstract: "young person"] OR [Abstract: kid*]] AND [[Publication Title: "mental health"] OR [Publication Title: "mental disorders"] OR [Publication Title: anxiety] OR [Publication Title: depressi*] OR [Publication Title: pyschotherap*] OR [Publication Title: phobia] OR [Publication Title: "phobic disorder*"] OR [Publication Title: "cognitive behavio* therapy"] OR [Publication Title: hci] OR [Publication Title: "human-computer interact*"] OR [Publication Title: "human computer interact*"] OR [Publication Title: educat*] OR [Publication Title: learning] OR [Publication Title: "behaviour change"] OR [Publication Title: or behav* or cbt or] OR [Publication Title: "cognitive behav* therapy"]] AND [Publication Date: (01/01/2010 TO 30/06/2021)] |
| IEEE Search String – (Dates limited to 01/01/2010 TO 30/06/2021) |
| ("All Metadata":cocreate OR "All Metadata":cocreation OR "All Metadata":co-create OR "All Metadata":codesign OR "All Metadata":co-design OR "All Metadata":particip* OR "All Metadata":“participatory design” OR "All Metadata":patient-cent* OR "All Metadata":patient-partner OR "All Metadata":co-produce OR "All Metadata":coproduction OR "All Metadata":user-cent* OR "All Metadata":collaborat* OR "All Metadata":cooperative OR "All Metadata":co-operative OR "All Metadata":iterative) AND ("All Metadata":Game OR "All Metadata":“Video Game” OR "All Metadata":videogame OR "All Metadata":“computer game” OR "All Metadata":Gami* OR "All Metadata":Game-based) AND ("All Metadata":“Mental health” OR "All Metadata":“Mental Disorders” OR "All Metadata":Anxiety OR "All Metadata":depression OR "All Metadata":pyschotherapy OR "All Metadata":phobia OR "All Metadata":“Phobic disorder” OR "All Metadata":“cognitive behavioural therapy” OR "All Metadata":"learning" OR "All Metadata":"behav*" OR "All Metadata":"behav* change" OR "All Metadata":education OR "All Metadata":CBT OR "All Metadata":"cognitive behav*" OR "All Metadata":HCI OR "All Metadata":"human computer interaction" OR "All Metadata":"human-computer interaction") AND ("All Metadata":Adolescent OR "All Metadata":Child OR "All Metadata":“Young people” OR "All Metadata":“young person” OR "All Metadata":kid) |
| Scopus Search String |
| ( TITLE-ABS-KEY ( cocreat*  OR  co-creat*  OR  codesign*  OR  co-design  OR  "participatory design"  OR  patient*-cent*  OR  patient*-partner*  OR  co-produc*  OR  coproduc*  OR  collabora*  OR  cooperat*  OR  co-operat*  OR  iterat* )  AND  TITLE-ABS-KEY ( game*  OR  "Video Game*"  OR  videogame*  OR  "computer game*"  OR  gami*  OR  game-based )  AND  TITLE-ABS-KEY ( "Mental health"  OR  "Mental Disorder*"  OR  anxiety  OR  depression  OR  psychotherap*  OR  phobia  OR  "Phobic disorder*"  OR  "cognitive behavio* therapy"  OR  "learning"  OR  "cognitive behavio* therapy"  OR  "CBT"  OR  "behavi*"  OR  "behavi* change"  OR  "education"  OR  "human computer interact*"  OR  hci  OR  "human-computer interaction" )  AND  TITLE-ABS-KEY ( adolescent  OR  child*  OR  "Young people"  OR  "young person"  OR  kid* ) )  AND  ( LIMIT-TO ( DOCTYPE ,  "ar" )  OR  LIMIT-TO ( DOCTYPE ,  "cp" )  OR  LIMIT-TO ( DOCTYPE ,  "re" ) )  AND  ( LIMIT-TO ( PUBYEAR ,  2021 )  OR  LIMIT-TO ( PUBYEAR ,  2020 )  OR  LIMIT-TO ( PUBYEAR ,  2019 )  OR  LIMIT-TO ( PUBYEAR ,  2018 )  OR  LIMIT-TO ( PUBYEAR ,  2017 )  OR  LIMIT-TO ( PUBYEAR ,  2016 )  OR  LIMIT-TO ( PUBYEAR ,  2015 )  OR  LIMIT-TO ( PUBYEAR ,  2014 )  OR  LIMIT-TO ( PUBYEAR ,  2013 )  OR  LIMIT-TO ( PUBYEAR ,  2012 )  OR  LIMIT-TO ( PUBYEAR ,  2011 )  OR  LIMIT-TO ( PUBYEAR ,  2010 ) )  AND  (  LIMIT-TO ( OA ,  "all" ) ) |
| Web of Science Search String – (Dates limited to 01/01/2010 TO 30/06/2021) |
| (cocreat*  OR co-creat*  OR codesign*  OR co-design  OR particip*  OR "participatory design"  OR patient*-cent*  OR patient*-partner*  OR co-produc*  OR coproduc*  OR user-cent*  OR collaborat*  OR cooperat*  OR co-operat*  OR iterat*) AND TOPIC: ("Mental health"  OR "Mental Disorders"  OR Anxiety  OR depressi*  OR pyschotherap*  OR phobia  OR "Phobic disorder*"  OR "cognitive behavio* therapy"  OR "education"  OR "human-computer inter*"  OR "HCI"  OR "Humam computer inter*"  OR "learning"  OR "CBT"  OR "behavi*") AND TOPIC: (Adolescent  OR Child*  OR "Young people"  OR "young person"  OR kid*) AND TOPIC: (Game*  OR "Video Game*"  OR videogame  OR "computer game*"  OR Gami*  OR Game-based) |
