# Supplementary material for: Children and Young People’s Involvement in Designing Applied Games: Scoping Review
Source: JMIR Serious Games. 2023 Mar 16;11:e42680. doi: 10.2196/42680 (PMC10131627; doi:10.2196/42680)
Supplement: Multimedia Appendix 3 [file games_v11i1e42680_app3.docx]

| **Authors** | **Self-Label** | **Druin's Taxonomy** | | | | **Capacity of involvement for primary end user** | **Study Goals** | **Activities** |
| --- | --- | --- | --- | --- | --- | --- | --- | --- |
|  |  | **User** | **Tester** | **Informant** | **Design Partner** |  |  |  |
| All et al. | codesigners / informants |  |  | X |  | Not Stated | Design a game with participants,Determine Features and Functionality, | (Focus) Group Discussion,Interviews (with end-users),Introductory media (presentation, movie etc),Map Task,Game ideation and discussion,Scenario Based Tasks,Blank template task |
| Alves & Hostins | null |  |  | X |  | Informant, co-designer, playtester and end user | Design a game with participants,Determine Features and Functionality, | Low-fidelity Prototyping,Scenario Based Tasks,Timeline (narrative design),Game-play Evaluation,Existing Games (demonstration or play),Existing Games (demonstration or play),Storyboarding,Paper Prototyping,(Focus) Group Discussion,Problem Solving,Brainstorming,Role-play (or simulation) |
| Al-Wabil et al. | validation | X | X | X | X | Co-designer | Determine Features and Functionality, | Interviews (with end-users),Gaze-Plots,Eyetracking Games |
| Anacleto et al. | null |  |  |  | X | Informant | Determine Features and Functionality, | Surveys or Questionnaires,Paper Prototyping,Timeline (narrative design),Brainstorming |
| Anthony et al. | Informant |  |  | X |  | Co-designer and Informant | Understand needs of end-user,Develop skills, | Surveys or Questionnaires,(Focus) Group Discussion |
| Aufegger et al. | informant |  |  | X | X | Co-designer | Understand perceptions and concerns,Determine Features and Functionality, | (Focus) Group Discussion,Surveys or Questionnaires,Timeline (narrative design) |
| Benton et al. | Informant |  |  |  | X | Informant | Understand perceptions and concerns,Design a game with participants, | Existing Games (demonstration or play),Feedback Session,LEGO team building,Paper Prototyping,Surveys or Questionnaires,Storyboarding,Visual Schedules |
| Benton & Johnson | null |  |  | X |  | Co-designer | Examine Involvement,Deliver content or skills development , | Visual Schedules,Surveys or Questionnaires,Paper Prototyping,Feedback Session |
| Bonsignore et al. | codesigner |  |  | X | X | Informant | Design a game with participants,Explore methodology, | Storytelling,Snack-time' Icebreaker,"Question of the day",Design Challenge,Sticky Notes,(Focus) Group Discussion,Game-play Evaluation,Scenario Based Tasks,Surveys or Questionnaires,Peer Interviews,Wireframe designs |
| Bossavit & Parsons | users and informants , designers ,testers |  |  | X |  | Validation | Examine involvement,Design a game with participants, | Existing Games (demonstration or play),Visual Schedules,Surveys or Questionnaires,Free-Play ,Paper Prototyping,Game-play Evaluation,Presentations,Playtesting |
| Cassidy et al. | informant |  |  |  | X | Not Stated | Feedback on end-product,Examine involvement,Understand perceptions and concerns | Design Packs,Role-play (or simulation),Low-fidelity Prototyping |
| Cheng et al. | Informant? |  |  | X |  | Informant | Feedback on end-product,Determine Features and Functionality, | Interviews (with end-users),Preference Elicitation,Existing Games (demonstration or play),Paper Prototyping,Interviews (with end-users) |
| Christie et al. | informants |  | X | X |  | Co-designer | Understand preferences,Feedback on end-product,Determine Features and Functionality | Playtesting,Interviews (with end-users) |
| Durl et al. | informant / codesigner |  |  |  | X | Validation | Understand needs of end-user,Examine involvement, | Existing Games (demonstration or play),Surveys or Questionnaires,Sensitization session,Paper Prototyping,Card sorting task |
| Eriksson et al. | playtesters |  |  |  | X | Informant | Explore methodology, | Playtesting,Interviews (with end-users),Free-Play ,Playtesting |
| Gennari et al. | co-designer |  |  | X | X | Informant | Design a game with participants,Examine involvement, | Existing Games (demonstration or play),(Focus) Group Discussion,Icebreakers,Surveys or Questionnaires,Interviews (with end-users) |
| Gonsalves et al. | informants / codesigners |  |  | X |  | Co-designer | Determine Features and Functionality,Understand needs of end-user,Understand preferences | (Focus) Group Discussion,Playtesting,Existing Games (demonstration or play),Storyboarding,Paper Prototyping,(Focus) Group Discussion |
| Kang et al. | Developer and motivators |  |  | X |  | Co-designer and Informant | Explore methodology,, | Existing Games (demonstration or play) |
| Kangas | player/ playtester | X |  |  |  | Validation | Develop skills,Design a game with participants, | Scenario Based Tasks,Role-play (or simulation),Free-Play ,Existing Games (demonstration or play),Blank template task,Feedback Session |
| Khaled & Vasalou | Co-designer/informant |  |  | X | X | Co-designer | Explore methodology,Examine involvement, | Brainstorming,Role-play (or simulation),Paper Prototyping,Storyboarding,Introductory media (presentation, movie etc) |
| Kostenius et al. | Informant |  |  | X | X | Not Stated | Explore methodology,, | (Focus) Group Discussion,Brainstorming,Movie creation task,Feedback Session,Logbooks (or taskbook, or Diaries) |
| Lee et al | informant |  |  |  | X | Co-designer and Informant | Facilitate context discussion,, | Existing Games (demonstration or play),Interviews (with end-users),Storyboarding,Q&A,Paper Prototyping,Blank template task |
| Leitao et al. | user, as a tester, and as an informant |  | X |  | X | Informant | Explore methodology,Examine involvement, | Surveys or Questionnaires,Free-Play ,Low-fidelity Prototyping,Paper Prototyping,Brainstorming |
| Malinverni et al | co-designer/ validation |  |  | X | X | Co-designer | Develop skills,Design a game with participants, | Introductory media (presentation, movie etc),Visual Templates,Existing Games (demonstration or play),Paper Prototyping,Role-play (or simulation),Storyboarding |
| Martens et al. | codesign / informant |  |  | X |  | Informant, co-designer, playtester and end user | High Quality Product,Understand preferences,Feedback on end-product | Playtesting,Free-Play ,Interviews (with end-users),Paper Prototyping,Logbooks (or taskbook, or Diaries) |
| Marti et al. | co-design |  |  |  | X | Informant | Design a game with participants,, | Storytelling,Cultural Probes,Storyboarding,Map Task,Paper Prototyping,3D Modelling activity |
| Metatla et al. | co-design | X |  | X |  | Co-designer and Informant | Understand perceptions and concerns,Examine involvement, | Activity Sheets,Paper Prototyping,Role-play (or simulation),Low-fidelity Prototyping,(Focus) Group Discussion,(Focus) Group Discussion |
| Nouwen et al. | co-designers |  |  | X |  | Co-designer and Validation | Develop skills,Examine involvement, | Interviews (with end-users),Playtesting,Logbooks (or taskbook, or Diaries),Interviews (with end-users),Introductory media (presentation, movie etc),Sensitization session,Assignments or Homework,Paper Prototyping,Low-fidelity Prototyping |
| Patchen et al. | informants |  |  | X | X | Informant | Feedback on end-product,Design a game with participants,Determine Features and Functionality | (Focus) Group Discussion,Playtesting,Brainstorming,User Stories (personas),Paper Prototyping |
| Pavarini et al. | co-researcher |  |  | X | X | Co-designer | Design a game with participants,, | Role-play (or simulation),Peer Interviews |
| Pollio et al | null |  |  | X |  | Informant | Design a game with participants,, | Storyboarding,Existing Games (demonstration or play),Toolkit (game design),Paper Prototyping |
| Porcino et al. | inclusion/validation. |  |  |  | X | Co-designer | Design a game with participants,Explore methodology, | Q&A |
| Powell et al. | validation | X |  |  |  | Informant | Determine Features and Functionality,Develop skills, | Interviews (with end-users) |
| Rötkönen et al. | informant / codesigner |  |  |  | X | Playtesters | Create Guidelines,, | Existing Games (demonstration or play),Playtesting,Existing Games (demonstration or play) |
| Raynes-Goldie & Allen | Codesigner |  |  | X |  | Informant, co-designer, playtester and end user | Develop skills,Design a game with participants, | Playtesting,Feedback Session,Storytelling,(Focus) Group Discussion |
| Regal et al. | Informants |  | X |  |  | Informant | Design a game with participants,Develop skills, | Building Blocks,Game design Workshops,Brainstorming,Interviews (with end-users) |
| Romero et al. | null | X | X | X | X | Informant | Develop skills,Determine Features and Functionality, | Existing Games (demonstration or play),Game design Workshops,Pitching (idea or prototype),Feedback Session |
| Stalberg et al | codesigner/informant |  |  | X |  | Informant | Determine Features and Functionality,Design a game with participants,Examine involvement | Interviews (with end-users),Paper Prototyping,Storyboarding |
| Sutton et al. | codesigners |  |  | X |  | Co-designer and Informant | Understand perceptions and concerns,Design a game with participants,Improved User Experience | (Focus) Group Discussion,Introductory media (presentation, movie etc),Sticky Notes |
| Terlouw et al. | ideation |  |  | X |  | Co-designer | Design a game with participants,Develop skills, | Brainstorming,Paper Prototyping,,Existing Games (demonstration or play),Sticky Notes |
| Triantafyllakos et al. | co-designer |  |  | X | X | Co-designer | Design a game with participants,Explore methodology, | Playtesting,Surveys or Questionnaires |
| Vallentin-Holbech et al. | co-designers/informants |  |  | X | X | Informant | Design a game with participants,Understand preferences, | Feedback Session,Role-play (or simulation),Introductory media (presentation, movie etc),Flow-charts,(Focus) Group Discussion,(Focus) Group Discussion,Playtesting,Interviews (with end-users),Storyboarding |
| Van Geit et al. | informant |  |  | X | X | Informant | Explore methodology,, | Interviews (with end-users),Brainstorming,Surveys or Questionnaires,(Focus) Group Discussion,Sticky Notes,Storyboarding |
| Vasalou & Khaled | informant |  |  | X |  | Co-designer and Informant | Determine Features and Functionality,Create Guidelines,Examine involvement | Interviews (with end-users),(Focus) Group Discussion,Role-play (or simulation) |
| Waddington et al. | informants |  |  | X |  | Co-designer and Informant | Explore methodology,Examine involvement,Facilitate context discussion | Playtesting |
| Werner-Seidler et al. | informant |  |  | X |  | Co-designer | Understand preferences,Design a game with participants, | (Focus) Group Discussion,Low-fidelity Prototyping,Icebreakers,Existing Games (demonstration or play),Mind Maps,Sticky Notes,Playtesting |
| Zhu et al. | codesign |  |  |  | X | Playtesters | Improved User Experience,Design a game with participants, | Low-fidelity Prototyping,Icebreakers,Existing Games (demonstration or play),Mind Maps,Sticky Notes,Playtesting |
